# Recovery from cold-induced mitochondrial fission in endothelial cells requires reconditioning temperatures of ≥ 25◦C

**DOI:** 10.3389/frtra.2022.1044551

**Published:** 2022-11-03

**Authors:** Leonard Quiring, Luisa Caponi, Dhanusha Schwan, Anja Rech, Ursula Rauen

**Affiliations:** ^1^Klinische Forschergruppe 117, Universitätsklinikum Essen, Essen, Germany; ^2^Institut für Physiologische Chemie, Universitätsklinikum Essen, Essen, Germany

**Keywords:** mitochondrial dynamics, mitochondrial fragmentation, mitochondrial fusion, mitochondria, endothelium, transplantation, machine perfusion

## Abstract

Mitochondrial integrity and function constitute a prerequisite for cellular function and repair processes. We have previously shown that mitochondria of different cell types exhibit pronounced fragmentation under hypothermic conditions. This fission, accompanied by a decline of cellular ATP content, showed reversibility at 37◦C. However, it is unclear whether other temperatures as currently discussed for reconditioning of organs allow this reconstitution of mitochondria. Therefore, we here study in a model of cultured porcine aortic endothelial cells how different rewarming temperatures affect mitochondrial re-fusion and function. After 48 h cold incubation of endothelial cells in Krebs-Henseleit buffer with glucose (5 mM) and deferoxamine (1 mM) at 4◦C pronounced mitochondrial fission was observed. Following 2 h rewarming in cell culture medium, marked fission was still present after rewarming at 10◦ or 15◦C. At 21◦C some re-fusion was visible, which became more marked at 25◦C. Networks of tubular mitochondria similar to control cells only re-appeared at 37◦C. ATP content decreased at 4◦C from 3.6 ± 0.4 to 1.6 ± 0.4 nmol/10^6^ cells and decreased even further when rewarming cells to 10◦ and 15◦C. Values after rewarming at 21◦C were similar to the values before rewarming while ATP gradually increased at higher rewarming temperatures. Metabolic activity dropped to 5 ± 11% of control values during 4◦C incubation and recovered with increasing temperatures to 36 ± 10% at 25◦C and 78 ± 17% at 37◦C. Integrity of monolayers, largely disturbed at 4◦C (large gaps between endothelial cells; cell injury ≤ 1%), showed partial recovery from 15◦C upwards, complete recovery at 37◦C. Endothelial repair processes (scratch assay) at 25◦C were clearly inferior to those at 37◦C. These data suggest that reconditioning temperatures below 21◦C are not optimal with regard to reconstitution of mitochondrial integrity and function. For this goal, temperatures of at least 25◦C appear required, with 30◦C being superior and 37◦C yielding the best results.

## Introduction

Machine perfusion has recently regained interest as a promising preservation technique for extended criteria donor organs to overcome organ shortage (1, 2). Hypothermic machine perfusion of kidneys and livers for example improved clinical outcomes in comparison with static cold storage (3, 4). Normothermic machine perfusion even allowed pre-transplantation viability assessment and repair (2, 5, 6). But with the requirement of safe, non-interrupted perfusion during organ transport, both methods are high in costs and complexity. Controlled oxygenated rewarming utilizes the advantages of conventional cold storage for transportation but enables organ reconditioning in the transplant center, where the organ is gradually rewarmed to avoid injury by rapid rewarming (7). The reconditioning approach offers the option for *ex vivo* treatment of the organ, tissue regeneration, viability assessment and immunomodulation of the graft prior to implantation into the recipient (8, 9). Even brief reconditioning periods of controlled oxygenated rewarming allowed for better energetic recovery and thus better graft function in kidneys and livers (7, 10). However, data on the optimal temperature at which organs should be reconditioned is limited (2, 8, 11, 12). Thus, cold (low potential for functional assessment and repair processes), subnormothermic (unclear potential for functional assessment and repair) and physiological temperatures (best potential for functional assessment and repair, but high oxygen requirement and disadvantage of full rewarming prior to the implantation procedure) are used (8). The scientific basis for optimizing temperature in reconditioning protocols, i.e., knowledge of the effects of different rewarming temperatures on the diverse cellular processes, is scarce.

During rewarming, injurious processes initiated during cold incubation are accelerated and result in rewarming injury (13–16). Furthermore, upon rewarming the abruptly increasing metabolism and energy demand in cells with decreased energy levels present after cold storage poses a difficult challenge for the cells (16–18). With their crucial role in energy provision, mitochondria are thus likely to be key to reconditioning and repair processes. However, mitochondria exhibit enhanced fragmentation at 4◦C, a process known as cold-induced mitochondrial fission (19, 20). This phenomenon could be seen in many different cell types (20–25). Evidence of mitochondrial fission was observed after cold ischemia/reperfusion of kidneys and livers (26–28), suggesting its occurrence also in whole organs. Cold-induced mitochondrial fission was recently characterized in porcine aortic endothelial cells (19) where it occurred as early as after 3 h at 4◦C and was associated with a decrease in ATP content. In healthy cells mitochondrial fission was reversible when cells were rewarmed to 37◦C. However, ATP recovery after cold incubation was compromised if re-fusion was prevented. Thus, re-fusion of mitochondria and the re-establishment of a normal mitochondrial network appear to be a prerequisite for adequate energy supply. Incomplete mitochondrial re-fusion after hypothermic storage is thus likely to compromise organ function and repair processes during reconditioning of organs. However, it is currently totally unclear, which temperature is required for the reconstitution of mitochondrial morphology and function after a cold storage period.

Therefore, we here assess in a cell culture model at which rewarming temperature mitochondrial re-fusion, ATP production and cellular regenerative capacity return after cold incubation.

## Materials and methods

### Isolation and culture of porcine aortic endothelial cells

For this study, we used porcine aortic endothelial cells because mitochondrial fission has already been characterized in these cells (19) and endothelial cells have been shown to be most sensitive to hypothermia (13, 29, 30). Moreover, as primary cells, they are less dedifferentiated and relatively close to the characteristics of endothelial cells *in vivo*, and they are primary endothelial cells that are available in sufficient quantities.

Porcine aortae were obtained from a local slaughterhouse and endothelial cells were isolated mechanically as described previously (31). Cells were cultured in M199 cell culture medium (Bio&Sell, Germany) supplemented with fetal calf serum (20% *v/v*), L-glutamine (2 mM) and antibiotics (100 U/ml penicillin, 100 U/ml streptomycin). They were used for experiments in a confluent state 2 days after splitting 1:3 as described previously (19).

### Cold incubation/rewarming

Cold incubation was performed as described previously (19) in modified Krebs–Henseleit (KH) buffer (143.6 mM Na^+^; 128.3 mM Cl^−^; 25.0 mM ${\text{HCO}}_{3}^{-}$; 5.9 mM K^+^; 1.2 mM Mg^2+^; 1.2 mM ${\text{SO}}_{4}^{2-}$; 1.2 mM H_2_${\text{PO}}_{4}^{-}$; 2.5 mM Ca^2+^; 20.0 mM HEPES) supplemented with glucose (5 mM) and deferoxamine (1 mM, Novartis, Basel, Switzerland), the latter added to prevent iron-dependent cellular and mitochondrial injury (13, 20). After cold incubation, cells were washed once with cold HBSS, cold cell culture medium was added and cells were slowly rewarmed to the different temperatures for 1–2 h (“rewarming period”). For some assays, this “rewarming period” was followed by a “re-culture period” of 48 h at normal cell culture conditions (37◦C) to simulate the return of a graft to 37◦C in the recipient. Cells were kept under a 5% CO_2_, 21% O_2_ and 74% N_2_ atmosphere (for the maintenance of the bicarbonate buffer system) at all temperatures.

### Cell morphology

For phase contrast microscopy including time lapse videos, the cells were grown on 6-well plates. Microscopy was performed using an Axio Observer.Z1 (with LD A-Plan 20x/0.30 Ph1, Zeiss, Germany) equipped with an incubator and a cooling/heating incubation insert P-Set 2000 (Pecon, Germany).

For the scratch assay, the cell monolayer was scratched with a sterile tip directly after cold incubation and cellular reaction during re-culture was documented using a time lapse video for 20 h. Control scratches were performed in cultures that had not been exposed to cold incubation. For technical reasons, time lapse videos with different rewarming temperatures (i.e., 25◦ and 37◦C) could not be performed in parallel, therefore a separate 37◦C control was set up for each rewarming temperature. For evaluation, each scratch was compared before and after the re-warming and re-culture period and scratch area was measured at selected time points (0, 1, 2, 3, 4, 5, 6, 7, 8, 12, 16, 20 h) using the ImageJ software. The slope of the linear portion of scratch reduction was determined. Slopes were normalized to the corresponding control.

### Mitochondrial morphology

Mitochondrial morphology was assessed as previously described (19). Cells were stained with MitoTracker Red CMXRos (150 nM; Invitrogen, Carlsbad, CA, USA) in KH buffer with glucose (5 mM) for 20 min at 37◦C. After washing, cells were incubated in glucose-containing KH buffer without dye for 2 h and then exposed to hypothermia as described above.

After hypothermia or after rewarming, the cells were fixated with paraformaldehyde (3.7% *w/v*) in cell culture medium for 10 min at the respective incubation temperature followed by 15 min at 37◦C, then mounted and analyzed by fluorescence microscopy (Axio Observer.Z1 with Apotome1, Plan-Apochromat 63x/1.40 Oil DIC, Zeiss, Germany; λ_exc._ = 546 ± 6 nm, λ_em._ ≥ 590 nm).

### ATP measurement

Cells were grown on 6-well plates and ATP content was determined after the different incubation conditions using the ATP Bioluminescence Assay Kit CLSII (Roche, Mannheim, Germany) according to the manufacturer's instructions.

### Metabolic activity

As parameter of metabolic activity the reduction of resazurin to resorufin was used. At the end of the experimental incubation, cells were washed and incubated in HBSS with 10 mM glucose for 15 min at the experimental incubation temperature. Thereafter 40 μM resazurin (Sigma-Aldrich, St. Louis, MO, USA) was added and resorufin formation was measured in a plate reader during the subsequent 20 min incubation at the respective incubation temperature (λ_exc._= 545 ± 20 nm, λ_em._= 600 ± 40 nm). Controls for all temperatures—without prior cold incubation—were included. Measurements were normalized to the corresponding warm control (measurement at 37◦C without prior cold incubation).

### Cell viability and proliferation

LDH activity was measured both in the supernatant and after cell lysis with 1% (*v/v*) Triton X-100 using a standard enzymatic assay based on pyruvate-dependent NADH oxidation (32). For assessment of cell injury extracellular, i.e., released, LDH activity was calculated as a percentage of total (i.e., released plus intracellular) LDH activity. In experiments involving re-culture periods, total LDH activity was additionally used as a marker of cell proliferation.

For the assessment of cell viability and cell number, cells were stained with Hoechst 33342 (1 μg/ml; Sigma-Aldrich, St. Louis, MO, USA) for 20 min and propidium iodide (5 μg/ml; Molecular Probes, Eugene, Oregon, USA) for 5 min at the end of the experimental incubation. Thereafter, cells were photographed by fluorescence microscopy and counted (Hoechst 33342: λ_exc._= 359 ± 24 nm, λ_em._= 445 ± 25 nm; propidium iodide: λ_exc._= 546 ± 6 nm, λ_em._≥ 590 nm; 5 randomly chosen fields of view per well).

For protein content, cells were lysed with RIPA buffer (17) supplemented with Protease-Phosphatase-Inhibitor (Cell Signaling, #5870S) at 4◦C for 30 min. Then the protein lysate was denatured in an ultrasonic bath and centrifuged at 17,000 × g for 15 min. Protein amount was measured by bicinchoninic acid (BCA) assay (Thermo Scientific, Waltham, MA, USA) at 562 nm.

Released glycocalyx components were assessed in the cell culture supernatants using the ELISA Kits for hyaluronic acid (MBS163202, MyBioSource, California, USA) and heparan sulfate (CSB-E09585h, Cusabio, Texas, USA) according to the manufacturers' instructions.

### Statistics

All experiments were performed in duplicate and repeated four times. Statistical analysis was performed with GraphPad Prism (GraphPad Software, San Diego, CA, USA). For analysis of the scratch assay, the Mann-Whitney test was used. For all other analyses the Friedman test was used.

## Results

### Mitochondrial morphology after cold incubation and during rewarming

**Figure 1:**
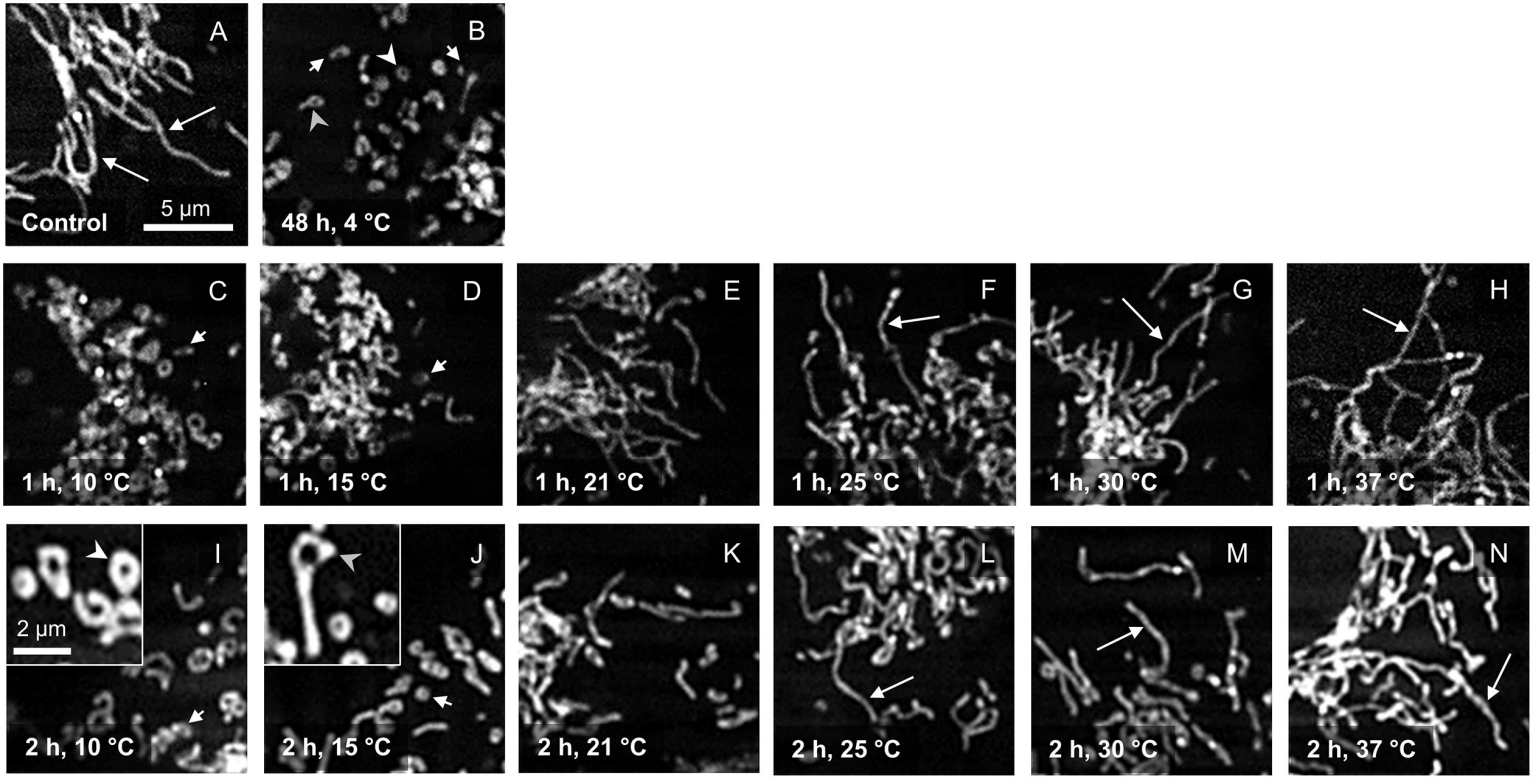
Mitochondrial morphology after cold incubation and during rewarming. Mitochondrial morphology of porcine aortic endothelial cells stained with MitoTracker Red (control; **A**) was assessed after 48 h cold incubation (4◦C; **B**) and after rewarming at temperatures from 10◦ to 37◦C for 1 h **(C–H)** and 2 h **(I–N)**. Tubular mitochondria are marked with a long arrow, while fragmented mitochondria are marked with a short arrow. Mitochondria with unusual morphologies such as donuts **(I)** and lassos **(J)** are shown enlarged in insets and are marked with white arrowheads (donuts) and gray arrowheads (lassos). Representative figures of *n* = 4 experiments.

In porcine aortic endothelial cells cultured at 37◦C, a network of long tubular mitochondria could be seen (Figure 1A). As described previously (19), extensive mitochondrial fragmentation was observed after incubation at 4◦C for 48 h (Figure 1B). This cold-induced mitochondrial fission was completely reversible during rewarming at 37◦C for ≥ 1 h (Figures 1H,N). Looking at the temperature dependence of this re-fusion, hardly any fusion of mitochondria was observed after “rewarming” at 10◦ and 15◦C for 1 h (Figures 1C,D). Even after 2 h of “rewarming,” only a slight re-fusion was present at 15◦C (Figure 1J). “Rewarming” at 10◦ and 15◦C rather led to unusual morphologies such as donuts and lassos (Figures 1I,J) as previously described for 4◦C (19). Longer mitochondria could be observed during rewarming at higher temperatures, starting at 21◦C (Figures 1E,K), more pronounced at 25◦ and 30◦C (Figures 1F,G). Especially when rewarmed for 2 h, the already clear re-fusion was even more marked at 25◦ and 30◦C (Figures 1L,M).

### ATP content after cold incubation and during rewarming

**Figure 2:**
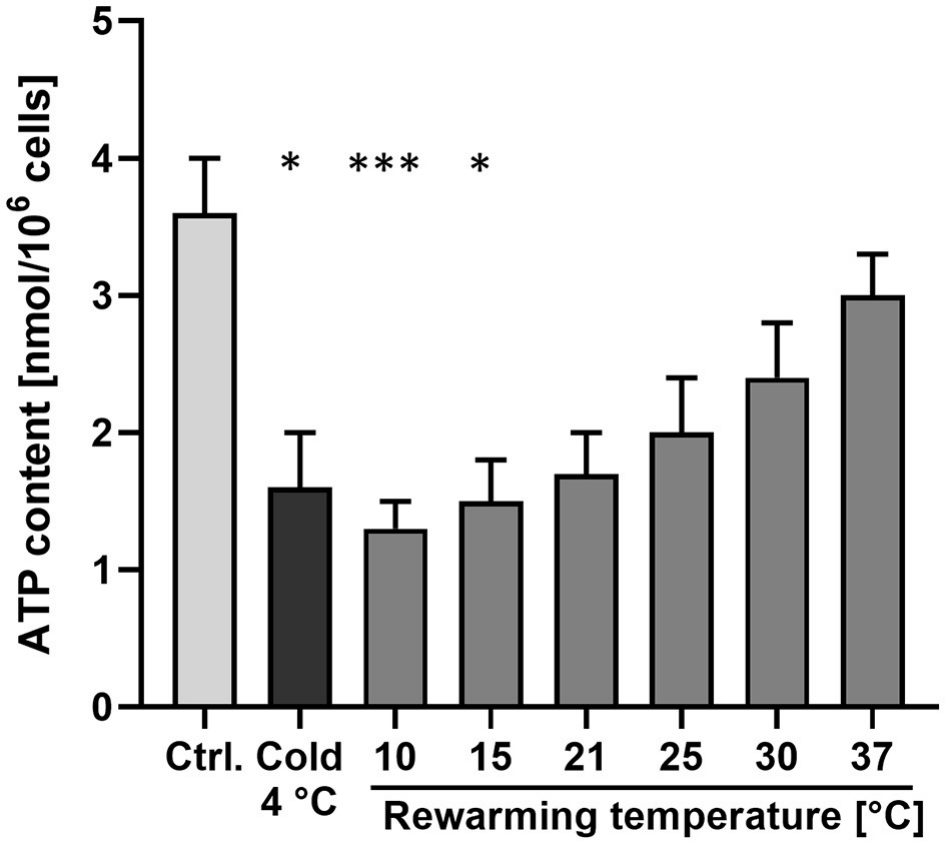
Cellular ATP content after cold incubation and during rewarming. Cellular ATP content of porcine aortic endothelial cells was measured under warm control conditions (Ctrl.), after 48 h at 4◦C and after rewarming at the indicated temperatures for 2 h. Means ± SD of *n* = 4 experiments. *Significant difference to the control (*p* ≤ 0.05); ***(*p* ≤ 0.001).

Cellular ATP content, i.e., the result of the central function of mitochondria, decreased significantly from 3.6 ± 0.4 to 1.6 ± 0.4 nmol/10^6^ cells after 48 h at 4◦C (Figure 2), matching the previous report (19), and largely returned to control levels after rewarming to 37◦C for 2 h. However, after “rewarming” at 10◦ and 15◦C, ATP content decreased even further compared to 4◦C (Figure 2). After 2 h rewarming at 21◦C, a similar ATP content as at 4◦C was measured. An increase in cellular ATP content could only be observed during rewarming at ≥ 25◦C. ATP content increased with increasing rewarming temperature.

### Metabolic activity after cold incubation

As metabolic activity itself is temperature-dependent, we measured cellular metabolic activity (resazurin reduction) at the different rewarming temperatures both in cells without prior cold incubation and during rewarming of cells that had been exposed to 48 h cold incubation at 4◦C.

**Figure 3:**
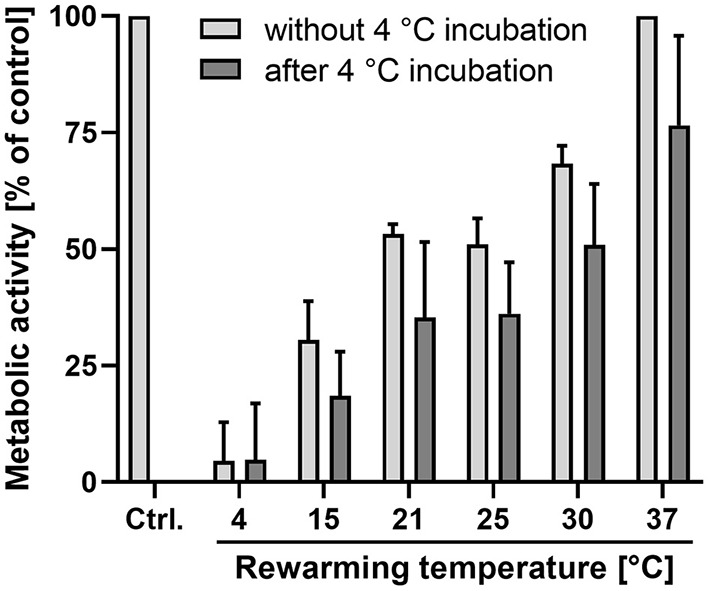
Metabolic activity at different temperatures without prior cold incubation and after cold incubation. Resazurin reduction was measured after 2 h incubation at the indicated temperatures without (light bars) and with (dark bars) prior cold incubation at 4◦C for 48 h. All values are given as percentage of the values for control cells (Ctrl.) not exposed to temperatures other than 37◦C. Means ± SD of *n* = 4 experiments.

Cells incubated at 4◦C showed expectedly a large reduction in metabolic activity (Figure 3), which was reduced to 5 ± 7% of that of the control cells at 37◦C. The other incubation temperatures also resulted in reductions in metabolic activity, amounting to 53 ± 2% at 21◦ and to 68 ± 3% of control values at 30◦C (Figure 3). After 48 h of cold incubation at 4◦C, the cells rewarmed to the different temperatures showed a similar temperature profile as the cells not exposed to 48 h cold incubation. However, the mean values of metabolic activity of rewarmed cells always remained below the respective value of cells not exposed to prior incubation at 4◦C. Thus, in cells rewarmed at 21◦C, 35 ± 15% of the control value was reached, in cells rewarmed at 30◦C it amounted to 51 ± 12%. Cells rewarmed at 37◦C reached 78 ± 17% of the control value.

### Cell morphology during rewarming

**Figure 4:**
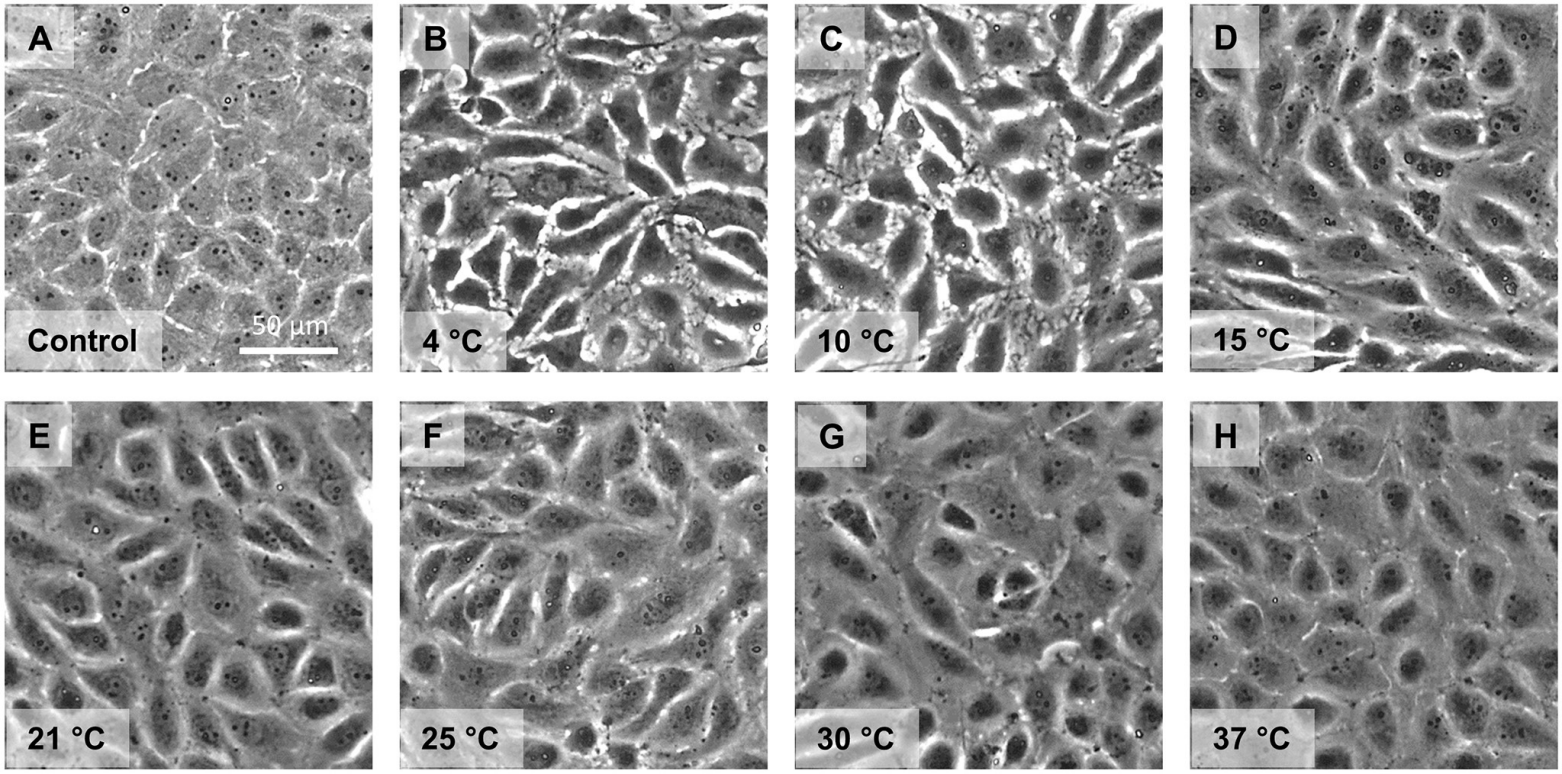
Monolayer morphology after cold incubation and during rewarming. Cell morphology of porcine aortic endothelial cells (Control, **A**) after cold incubation for 48 h at 4◦C **(B)** and after subsequent rewarming at the indicated temperatures for 2 h **(C–H)** was assessed by phase contrast microscopy. Representative figures of *n* = 4 experiments.

Cell morphology and monolayer integrity were judged using phase contrast microscopy. Control cells showed a confluent monolayer (Figure 4A). Endothelial cells incubated for 48 h at 4◦C exhibited marked intercellular gaps (Figure 4B). These gaps were still present after 2 h “rewarming” at 10◦C (Figure 4C). During “rewarming” at 15◦C the gaps started to close (Figure 4D). They were substantially closed after rewarming at 21◦, 25◦, and 30◦C (Figures 4E–G), completely closed after rewarming to 37◦C (Figure 4H).

### Closure of endothelial wounds during reconditioning at intermediate and physiological temperature

**Figure 5:**
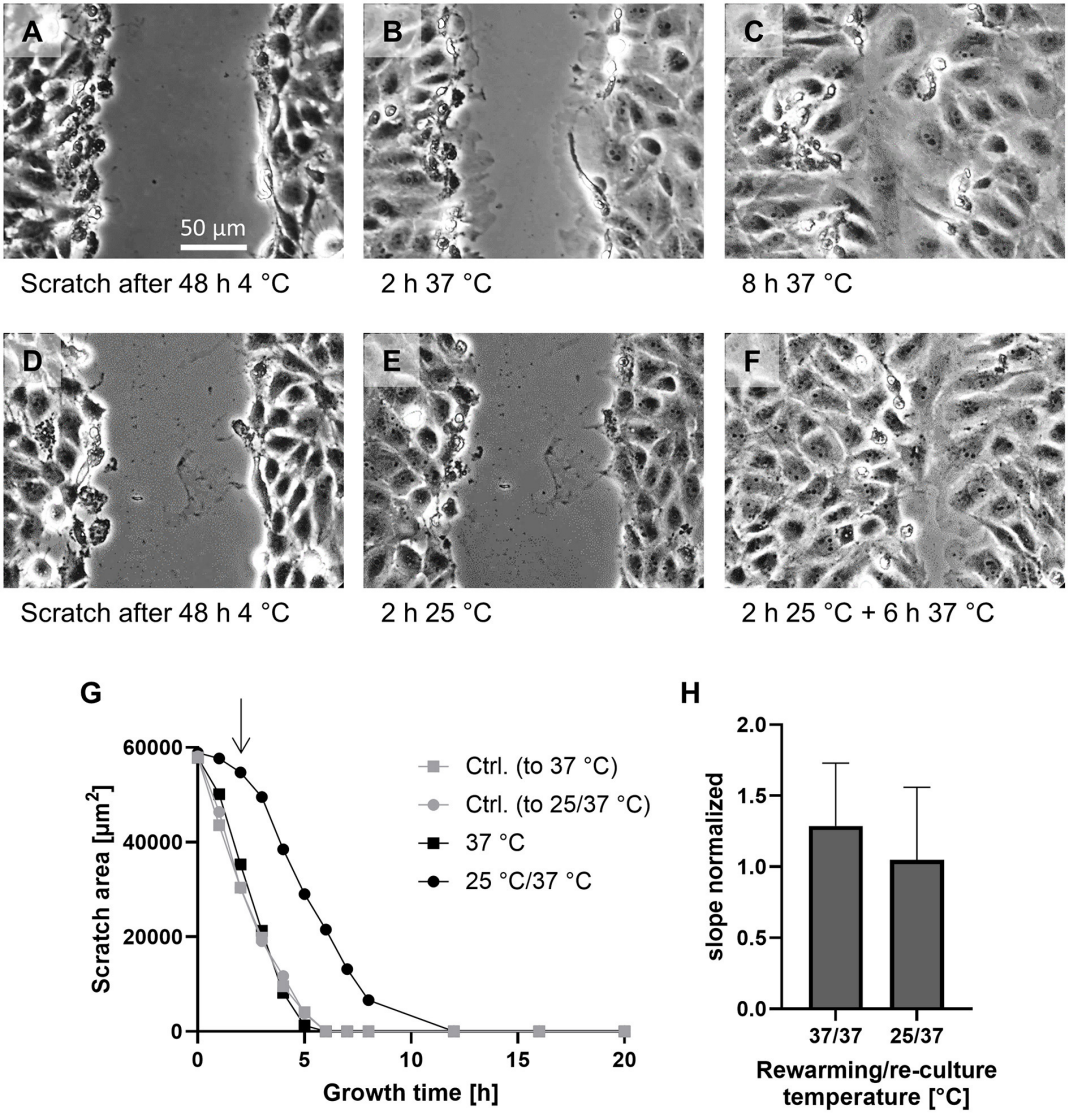
Scratch assay for assessment of cellular capacity for the repair of endothelial monolayer integrity. A scratch was introduced into the monolayer not exposed to hypothermia (Ctrl.) and into monolayers after cold incubation for 48 h at 4◦C and cultures were incubated at 37◦C or, for reconditioning, for 2 h at 25◦C and then at 37◦C. Images shown after 48 h at 4◦C **(A,D)**, after 2 h at 37◦C **(B)** or 25◦C **(E)** and 6 h re-culture at 37◦C **(C,F)**. Representative figures of *n* = 4 experiments. Scratch size was evaluated hourly from 0 to 8 h and every 4 h thereafter. Plot of scratch size under the indicated conditions of a representative experiment **(G)**. The arrow marks the switch from 25◦C reconditioning to 37◦C re-culture (filled circles). The slope in the linear region of the graphs of four experiments was normalized to the respective control **(H)**; mean ± SD of the normalized slopes are given.

To assess how healing of injuries in the endothelial monolayer was affected by intermediate vs. physiological reconditioning temperatures, a scratch assay was performed. The scratch was introduced directly after cold incubation (Figures 5A,D). Then, for comparison of reconditioning protocols, cold incubated cells were directly rewarmed to 37◦C (Figures 5B,C) or rewarmed to an intermediate temperature of 25◦C for 2 h (Figure 5E) followed by re-culture at 37◦C (Figure 5F). Controls were scratched without being exposed to cold incubation. Cells rewarmed at 37◦C showed marked ruffles at the proliferating edges after 2 h (Figure 5B, Supplementary Data 1). At 37◦C the decrease in scratch area was linear between hours 1–3 of rewarming (Figure 5G) and scratches were completely closed after 6–7 h of rewarming at 37◦C. Overall, scratch closure during rewarming at 37◦C was slightly more rapid than in controls not exposed to hypothermia (Figure 5G) as indicated by a normalized slope > 1 (Figure 5H).

Cells rewarmed at 25◦C showed hardly any ruffles while being at this temperature (Figure 5E, Supplementary Data 2) and hardly any decrease in scratch size during 25◦C incubation (Figure 5G). During the first 2 h of subsequent 37◦C re-culture, some large ruffles (less than after 37◦C rewarming) appeared (Supplementary Data 2) and the cells progressively moved into the scratch area and thus the rate at which the scratch area closed increased (Figure 5G) to a rate similar to control cells (normalized slope of 1.05 ± 0.48; Figure 5H), finally yielding a relatively homogeneous scratch closure (Figure 5F). Thus, cells rewarmed at 25◦C followed by 37◦C re-culture showed delayed scratch closure that did not occur until re-culture.

### Cell proliferation at different reconditioning temperatures

**Figure 6:**
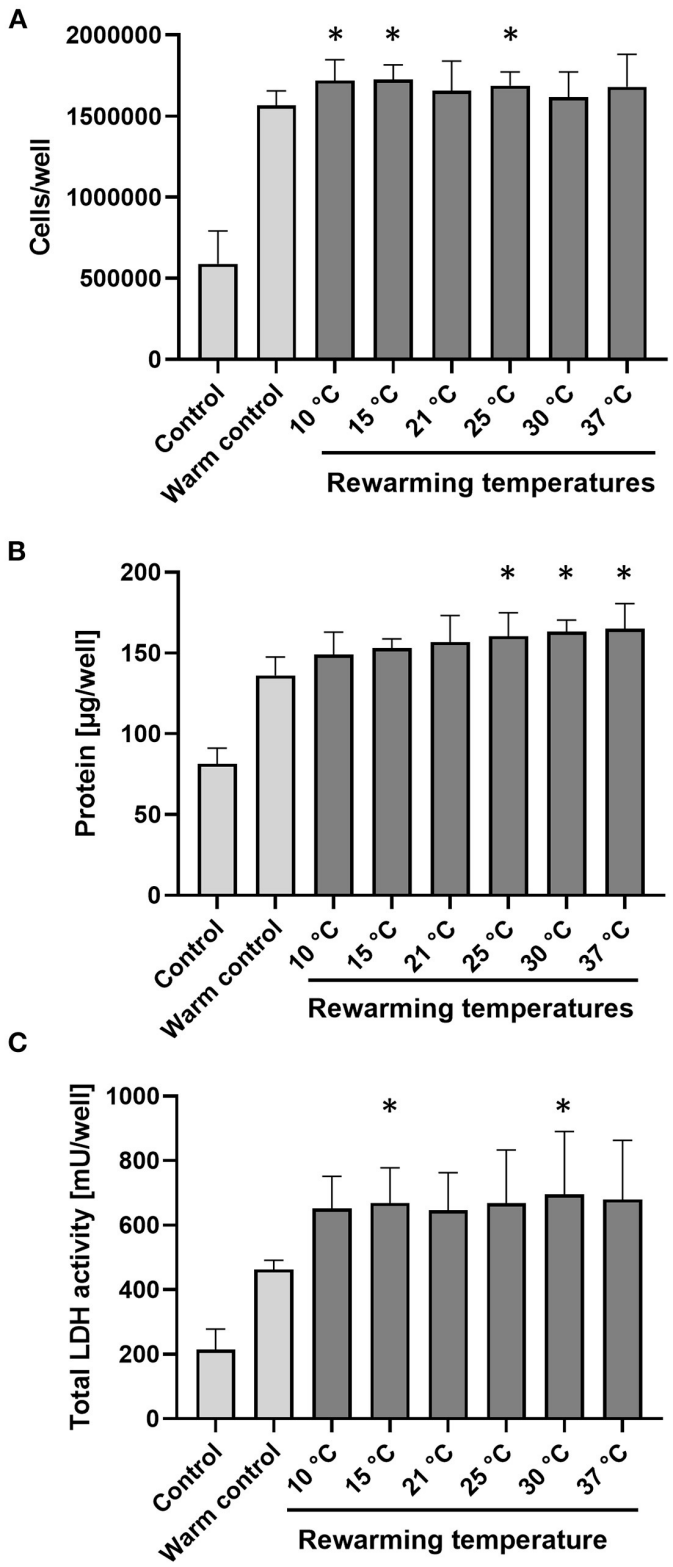
Proliferation of cells during re-culture following cold incubation and rewarming. Porcine aortic endothelial cells (control; indicates cells at time zero) were incubated for 48 h at 4◦C, rewarmed at indicated temperatures for 2 h and then re-cultured at 37◦C for 48 h. The warm control was cultured for 48 h at 37◦C without prior exposure to hypothermia. Proliferation is shown by number of cells **(A)**, protein amount **(B)**, and total LDH activity **(C)** per well. Means ± SD of *n* = 4 experiments. *Significant difference to control (*p* ≤ 0.05).

During 48 h re-culture subsequent to 48 h cold incubation and 2 h rewarming at the different rewarming temperatures, all cells proliferated as assessed by cell number, protein content and total LDH activity of the cultures (Figures 6A–C). Proliferation of cold-incubated, rewarmed cells was at least equal to that of control cells not exposed to hypothermia but cultured for 48 h (warm control): Cell numbers of re-cultured cells were marginally higher than cell numbers of warm controls (Figure 6A) and protein content of re-cultured cells was slightly higher than that of warm controls (Figure 6B). For total LDH values, values of re-cultured cells were about 45% higher than those of warm control cells (Figure 6C). For all three parameters, the temperature during the 2 h rewarming period only had minor effects on cell proliferation.

### Cell injury and release of glycocalyx components

Cell injury was low throughout all conditions (<1.2% LDH release after cold incubation, <3% after rewarming); even after 48 h re-culture injury remained below 7% without significant differences between the different rewarming temperatures (Table 1).

**Table 1:** Cell injury in endothelial cells after cold incubation and subsequent rewarming.

**Condition/Temperature**	**Propidium iodide uptake (%)**	**LDH release (%)**
Control	0.6 ± 0.4	-
Warm control	1.6 ± 0.9	0.6 ± 0.5
48 h, 4◦C	0.6 ± 0.5	0.4 ± 0.4
Rewarming 10◦C + re-culture	3.2 ± 0.7	5.9 ± 3.7
Rewarming 15◦C + re-culture	3.7 ± 1.6	6.3 ± 4.6
Rewarming 21◦C + re-culture	3.7 ± 1.5	4.5 ± 2.9
Rewarming 25◦C + re-culture	4.6 ± 2.7	5.0 ± 3.4
Rewarming 30◦C + re-culture	2.8 ± 0.8	5.4 ± 3.9
Rewarming 37◦C + re-culture	1.8 ± 0.3	4.9 ± 3.3

Cell injury was assessed after staining with Hoechst 33342 and propidium iodide and by determination of the release of cytosolic lactate dehydrogenase (LDH) from porcine aortic endothelial cells (Control, time zero) after 48 h at 37◦C (Warm control), after 48 h at 4◦C and after subsequent rewarming for 2 h at the indicated temperatures followed by 48 h re-culture.

Means ± SD of n = 4 experiments.

Assessment of the release of glycocalyx components showed that hyaluronic acid was released in basal amounts during normothermic incubation of control cells (Table 2). During cold incubation, the shedding of hyaluronic acid increased (Table 2) and in addition, a release of heparan sulfate was observed [17 ± 2 ng/cm^2^ endothelium during 48 h at 4◦C; <7 ng/cm^2^ endothelium during 48 h at 37◦C (and during 2 h rewarming)]. During 2 h rewarming after cold incubation the total release of hyaluronic acid was similar to the release during 48 h warm incubation of controls suggesting a far higher release rate during rewarming (Table 2) without significant differences between rewarming temperatures.

**Table 2:** Shedding of hyaluronic acid during cold incubation and rewarming.

	**Hyaluronic acid release**	
**Condition/Temperature**	Total [ng *(cm^2^ endothelium)^−1^]	Hourly release rate [ng *(cm^2^ endothelium*h)^−1^]
Warm control (48 h)	4.7 ± 0.8	0.1 ± 0.0
48 h, 4◦C	10.8 ± 0.2	0.2 ± 0.0
Rewarming 10◦C (2 h)	4.1 ± 0.1	2.1 ± 0.1
Rewarming 25◦C (2 h)	6.6 ± 0.5	3.3 ± 0.2
Rewarming 30◦C (2 h)	5.8 ± 1.4	2.9 ± 0.7
Rewarming 37◦C (2 h)	5.3 ± 0.5	2.7 ± 0.3

Hyaluronic acid was detected by ELISA in supernatants from porcine aortic endothelial cells after 48 h at 37◦C (Warm control), 48 h at 4◦C and rewarming for 2 h at the indicated temperatures. Total release was measured and hourly release rate was calculated for better comparability.

Means ± SD of n = 4 experiments.

## Discussion

The results obtained here show that re-fusion of mitochondria after cold incubation occurred progressively at ≥ 21◦C, while mitochondrial function came back successively at ≥ 25◦C suggesting that, from a mechanistical point of view, temperatures of ≥ 25◦C, even better of ≥ 30◦C are preferable for reconditioning.

Mitochondrial fission and fusion are physiological processes needed to adjust energy provision to energy demand (33), to ensure distribution of mitochondria to daughter cells (34) and to allow mitochondrial quality control and mitophagy (35, 36). While for the latter processes fission is required, mitochondrial fusion yields mitochondria that are more efficient in energy production (36). The increasingly higher ATP levels of cells rewarmed at ≥ 21◦C (Figure 2) with progressively fused mitochondria (Figure 1) goes in line with this latter notion.

Under normothermic conditions, mitofusins 1 and 2 together with optic atrophy protein 1 (Opa1) are the major factors responsible for mitochondrial fusion (37–39). Dynamin-related protein 1 (Drp1) is regarded to play the key role in fission together with its receptors fission 1 protein, mitochondrial fission factor, and mitochondrial dynamics proteins 49 and 51 (40–42). Under physiological conditions, both, fission and fusion, are highly regulated; fission, in particular, is activated by phosphorylation of Drp1 at S616.

Cold-induced mitochondrial fission was very pronounced (Figure 1), as described previously (19, 20), and has been observed in many cell types relevant for transplantation medicine, such as vascular endothelial cells (19, 20), hepatocytes (21, 25), corneal endothelial cells (22), kidney epithelial cells (23, 24), and renal tubules (17) and there is evidence for its occurrence in mouse and rat kidneys (26, 27) as well as human livers (28). However, in these whole organs, mitochondrial fission is not yet unequivocally proven because mitochondrial length determined by transmission electron microscopy, as in these studies, always has the inherent uncertainty of the section plane. Furthermore, in the whole organ studies, the described mitochondrial fragmentation occurring after cold ischemia/reperfusion cannot be attributed to hypothermia alone as hypothermia, ischemia, preservation solution and reperfusion might contribute. Cold-induced mitochondrial fission, as studied in cultured cells, is mechanistically not yet fully understood as it appears to occur in a at least partially Drp1-independent manner (19). In cultured endothelial cells cold-induced mitochondrial fission has been described to occur at temperatures ≤ 15◦C, and was most marked at 4◦C, i.e., in the temperature range commonly used for organ preservation; however, it was so far unclear at what temperature re-fusion occurs after mitochondrial fragmentation at 4◦C.

Here, we observed that at low “rewarming” temperatures of 10◦ and 15◦C, hardly any re-fusion of mitochondria occurred (Figure 1). There are a number of possibilities for the lack of fusion: lack of mitofusins 1 and 2, the GTPase known to mediate outer mitochondrial membrane fusion, cleavage of Opa1, the GTPase mediating inner mitochondrial membrane fusion [which is known to be processed by Oma1 (36)], lack of GTP for these GTPases, low activity of the GTPases at low temperatures, rigidity of the inner and outer mitochondrial membrane and/or lack of mitochondrial motility at the low temperatures. While the fact that rapid mitochondrial fusion was possible at higher rewarming temperatures (Figures 1F–H) renders insufficient levels of the fusion proteins mitofusins 1 and 2 and Opa1 after 48 h cold incubation unlikely, the other reasons all might contribute to low fusion activity. The unusual morphologies observed after “rewarming” at 10◦ and 15◦C, i.e., donut and lasso structures (Figures 1I,J), however, have been described to be a result of auto-fusion of mitochondria, either end-to-end (yielding donuts) or end-to-side (yielding lasso structures) (43). Their occurrence thus might suggest that limited fusion processes can occur at 10◦ and 15◦C and render limited mitochondrial motility at low temperature, required for mitochondria to encounter each other to allow fusion, a likely contributing factor. Mitochondria move, with the aid of dynamin and kinesin motor complexes, along microtubules (44), and mammalian microtubules are well-known to disintegrate at low temperature (45).

Exchange of metabolites, Ca^2+^ and mitochondrial DNA are considered to contribute to the higher efficiency of fused long mitochondria/mitochondrial networks in ATP production (36). Therefore, it is not surprising that the unusual auto-fused mitochondria at 10◦ and 15◦C did not lead to increases in ATP levels (Figure 2). ATP rather decreased at these temperatures, a finding that is likely explained by increasing metabolism, i.e., ATP consumption, in the presence of low ATP production.

At ≥ 21◦C mitochondria increasingly fused and ATP levels progressively increased reconstituting a normal mitochondrial network and reaching about 85% of pre-cold-storage ATP values after rewarming at 37◦C. However, it is important to note that return to 37◦C does not always lead to normalization of mitochondrial morphology and function: Dependent on cold incubation/storage time, the solution used for cold incubation and/or the presence of inhibitors of cold-induced injury, re-fusion of mitochondria was either possible or not (17, 20, 22, 25). Re-fusion was hampered under conditions under which cold-induced cell injury occurred (20, 22, 25), pointing to the requirement of “healthy cells”; otherwise fragmented mitochondria remained and subsequently underwent mitochondrial ultra-condensation or swelling and loss of membrane potential.

Cell damage was low in our model used here (Table 1) due to the addition of the iron chelator deferoxamine. Cold-induced injury and a major part of rewarming injury are iron dependent (13, 21, 46–48) and endothelial cells are known to be very sensitive to cold-induced injury and rewarming injury (13, 30, 49). In particular, iron-dependent injury has been shown to lead to mitochondrial permeability transition during rewarming (21, 50). To study the temperature dependence of mitochondrial dynamics and function during rewarming, this iron-dependent injury was inhibited. Furthermore, Krebs-Henseleit solution was used for cold incubation, although it is not used for whole organ preservation. However, there is evidence of a certain toxicity of preservation solutions (51), and University of Wisconcin solution has previously been shown to contribute to endothelial injury (20, 52). As, in this study, we wanted to characterize the temperature effects, not superimposed by any preservation solution toxicity, we chose to use Krebs-Henseleit buffer. Studies in complex settings with multiple injurious factors in parallel, such as temperature, hypoxia and preservation solution, can only contribute to knowledge after the individual processes have been understood.

Although cell damage was low throughout all conditions in our study (Table 1), we nevertheless observed some functional compromises at least in the first hours after rewarming. Cellular ATP content and metabolic activity (Figure 3) were always lower after cold incubation than without cold incubation. Furthermore, the release of glycocalyx components during hypothermia and especially during rewarming also suggest a functionally impaired endothelium (Table 2). Endothelial glycocalyx shedding has recently gained attention in organ preservation and reconditioning (53, 54). In addition, during cold incubation intercellular gaps in the endothelial monolayer appeared (Figure 4). This gap formation has also been described for corneal endothelial cells, lung epithelial cells and proximal tubular cells (22, 55, 56) and, in tissue, are likely to give rise to increased permeability. During rewarming, gap size decreased at ≥ 15◦C but complete closure was only observed at 37◦C (Figure 4H), similar to closure of an endothelial wound not occurring during rewarming at 25◦ but at 37◦C (Figure 5). Whether this is related to the lower ATP levels (Figure 2) or to the temperature dependence of cytoskeletal alterations or repair processes is currently unclear.

During re-culture, cell proliferation — a complex cellular function — was not impaired, regardless of the rewarming temperature (Figure 6). Protein levels appeared to be even slightly increased and LDH activity induced. While this suggests little late endothelial injury irrespective of the rewarming temperature, this finding might not be transferable to other cells types: Endothelial cells are very resistant to hypoxic or energy deficiency injury (57) and thus likely tolerate the low ATP levels occurring at rewarming to 10–15◦C (Figure 2) better than other cell types would.

Although endothelial cell proliferation and closure of wounds in the endothelial monolayer during re-culture at 37◦C were not affected by the temperature during the prior rewarming period (Figures 5, 6), insufficient gap closure/injury repair during the rewarming, i.e., reconditioning period, would, in a graft, give rise to oedema formation and unshielded sub-endothelial matrix, to which, upon reperfusion with blood, coagulation factors and platelets might attach and lead to microcirculatory deterioration and inflammation (58). Thus, the finding that endothelial proliferation during re-culture was not affected by initial rewarming temperature does not necessarily mean that rewarming temperature does not affect later processes.

We here provided, in an endothelial cell culture model, first mechanistic insights into the effects of different rewarming temperatures on the integrity and function of mitochondria in endothelial cells previously exposed to hypothermia. These data need to be confirmed with other cell types and whole organ models such as precision cut tissue slices or organoids, i.e., experimental models in which cells have an almost normal environment and interaction, and finally in whole organ and in transplantation experiments. Thereafter, these results need to be combined with the temperature dependences of further processes regarded to be important for organ quality. With a better understanding of the cellular processes, an optimization and refinement of reconditioning protocols for the different organs could be achieved. While for the study of such mechanistic processes cell culture models are indispensable, results need to be confirmed in tissue/organ models with the complex interplay of the different cell types and finally derived temperatures/protocols inevitably need to be confirmed in complex animal models of organ transplantation.

In conclusion, we here obtained data in an endothelial cell model suggesting that for optimal recovery of mitochondrial network and function during reconditioning after cold storage rewarming temperatures of ≥ 25◦C, best of 37◦C are preferable from a mechanistic point of view, a finding that now awaits confirmation in tissue/organ models.

## Data availability statement

The raw data supporting the conclusions of this article will be made available by the authors, without undue reservation.

## Author contributions

UR conceptualized the study. LQ, LC, DS, and AR conducted the experiments. LQ analyzed the data. LQ and UR contributed to interpretation of the data and wrote the paper, all other authors critically revised it. All authors read and agreed to the final version of the manuscript.

## Funding

We acknowledge support by the Open Access Publication Fund of the University of Duisburg-Essen.

## Conflict of interest

The authors declare that the research was conducted in the absence of any commercial or financial relationships that could be construed as a potential conflict of interest.

## Publisher's note

All claims expressed in this article are solely those of the authors and do not necessarily represent those of their affiliated organizations, or those of the publisher, the editors and the reviewers. Any product that may be evaluated in this article, or claim that may be made by its manufacturer, is not guaranteed or endorsed by the publisher.
